# Miller-Fisher Syndrome: A Case Report and Review of the Literature

**DOI:** 10.5811/cpcem.2020.7.48507

**Published:** 2020-09-09

**Authors:** Justina Truong, Jeff Conley, John Ashurst

**Affiliations:** Kingman Regional Medical Center, Department of Emergency Medicine, Kingman, Arizona

**Keywords:** Miller-Fisher syndrome, Guillain-Barré syndrome, neurological emergencies

## Abstract

**Introduction:**

Neurological complaints are a common presenting symptom seen by the emergency physician. However, the Miller-Fisher variant of Guillain-Barré syndrome is a rare cause of neurological complaints seen in the emergency department.

**Case Report:**

A 26-year-old male presented with dysphonia and bilateral hand and feet paresthesia after a recent diarrheal illness. Examination revealed the absence of tricep, brachioradialis, patellar and Achilles tendon reflexes bilaterally, and difficulty with phonation. Lumbar puncture revealed the presence of anti-GQ1b antibodies, and the patient was diagnosed with Miller-Fisher variant of Guillain-Barré.

**Discussion:**

Miller-Fisher syndrome is an acute, autoimmune response that typically follows either an upper respiratory or diarrheal illness. Typically associated with dysfunction of cranial nerves three, four, and six, Miller-Fisher syndrome may present with facial paralysis, opthalmoplegia, arefexia, or ataxia. Lumbar puncture with the presence of anti-GQ1b antibodies is indicative. Treatment could include supportive respiratory care, intravenous immunoglobulin therapy, or plasmapheresis.

**Conclusion:**

Miller-Fisher syndrome is a rare form of Guillain-Barré syndrome that the emergency provider should include in the differential when faced with a patient with cranial nerve dysfunction.

## INTRODUCTION

Neurological conditions account for approximately 5% of all emergency department (ED) visits annually and mostly include the diagnoses of headache, dizziness, weakness, and seizures.^1^ Rarely, however, does the emergency provider evaluate a patient with either bilateral descending paralysis or ophthalmoplegia. Miller-Fisher syndrome, a variant of Guillain-Barré syndrome, is characterized by the triad of areflexia, ataxia, and ophthalmoplegia and can rapidly progress to respiratory failure.^2^ We present a case of Miller-Fisher syndrome in a 26-year-old male following a diarrheal illness.

## CASE REPORT

A 26-year-old male presented to the ED due to four days of progressive changes in his voice with associated paresthesia in his hands and feet. He stated that when he drank liquids he had difficulty swallowing and had reflux of liquids out of his nasal passage. He did report a recent trip to India followed by several days of diarrhea, which had resolved several weeks earlier. He also noted difficulty with his vision but denied any weakness, difficulty with ambulation, or recent upper respiratory tract infections. His past medical history was negative, and he took no medications on a daily basis.

Physical examination revealed a healthy appearing male in no acute distress. When attempting to phonate, he had decreased motion of the soft palate and pooling of salvia in the oropharynx. His voice was markedly altered when asked to phonate. Neurologic exam revealed 5/5 muscle strength in the upper and lower extremities but absent triceps, brachioradialis, patellar and Achilles tendon reflexes. Subjective paresthesias were also noted in the hands and feet bilaterally with difficulty discriminating two points.

Complete blood count, basic metabolic profile, and magnesium levels were all normal. Computed tomography of the head was also without abnormality. The patient underwent lumbar puncture and was found to have a glucose of 56 milligrams per deciliter (mg/dL) (reference range 40–70 mg/dL), protein 25 mg/dL (reference range 15–45 mg/dL), and no bacterial growth on culture, but he had a positive anti-GQ1b antibody consistent with Guillain-Barré syndrome. Based upon his symptoms and clinical findings he was diagnosed with the Miller-Fisher variant of Guillain-Barré. He subsequently underwent intravenous (IV) immunoglobulin G (IgG) therapy and had complete resolution of his symptoms after six days of treatment.

## DISCUSSION

First described in 1984 by Phillips and Anderson, Miller-Fisher syndrome is a rare variant of Guillain-Barré syndrome and accounts for between 1–5% of all cases in western countries but 15–25% of all cases in Asia.^2^,^3^ With a mean age of onset of 43.6 years, Miller-Fisher syndrome affects males twice as frequently as females annually.^2^,^3^ Typically preceded by either an upper respiratory or diarrheal infection, the disease has been associated with the cytomegalovirus, Epstein-Barr virus, and *Campylobacter jejuni*.^3^,^4^

Although presentation can vary, the majority of patients will present with a form of distal paresthesia coupled with dysfunction of the cranial nerves.^3^,^4^ The classic triad of areflexia, ataxia, and ophthalmoplegia is not seen in every patient and is dependent upon the course and duration of illness.^3^,^4^ Neurologic symptoms typically will not present until 8–10 days following an illness and nadir six days following the initial presentation.^5^ Physical examination may reveal facial paresis, distal hyporeflexia and loss of vibratory and light-touch sensation in the distal extremities.^3^–^5^ Bilateral dilated pupils and pharyngeal involvement may also occur in patients with Miller-Fisher syndrome in the absence of other neurologic symptoms.^5^

Although the clinical features are indicative of the disease, lumbar puncture can further aid the emergency provider. The combination of a normal cell count with increased protein in the cerebral spinal fluid is classic, but normal protein levels do not essentially rule out the disease.^3^ The presence of IgG autoantibodies to GQ1b in the cerebral spinal fluid is strongly associated with Miller-Fisher syndrome and should be obtained to further aid the clinician to rule in or out the diagnosis.

ED management should be aimed at symptomatic care and respiratory support if needed. IV and oral steroids are no longer recommended in the course therapy and may actually slow recovery. IV immunoglobulin and plasma exchange therapy are now the standard of care for those with Miller-Fisher syndrome. Following treatment, the mortality is less than 5%, but recurrence of the disease can occur between 5–10% of the time.

CPC-EM Capsule

What do we already know about this clinical entity?

*Miller-Fisher syndrome is a variant of Guillain-Barre syndrome that presents with distal paresthesia coupled with dysfunction of the cranial nerves*.

What makes this presentation of disease reportable?

*Although presenting after a diarrheal illness, the patient did not have the classic triad of areflexia, ataxia, and ophthalmoplegia on examination*.

What is the major learning point?

*Miller-Fisher syndrome is diagnosed by the presence of immune globulin G autoantibodies to GQ1b in the cerbral spinal fluid and treatment includes intravenous immunoglobulin and plasma exchange therapy*.

How might this improve emergency medicine practice?

*A high index of suspicion and early respiratory support can prevent long term sequelae from complications associated with Miller-Fisher syndrome*.

## CONCLUSION

Although rarely seen by the emergency care provider, Miller-Fisher syndrome should be included in the differential diagnosis of patients who present with cranial nerve dysfunction or descending paralysis following a recent illness. When considered, lumbar puncture should be performed in the ED and treatment should be initiated after consultation with neurology to prevent progression of the disease.
